# Incidence and predictors of post-stroke cognitive impairment among patients admitted with first stroke at tertiary hospitals in Dodoma, Tanzania: A prospective cohort study

**DOI:** 10.1371/journal.pone.0287952

**Published:** 2024-04-10

**Authors:** Baraka Alphonce, John Meda, Azan Nyundo

**Affiliations:** 1 Department of Internal Medicine, School of Medicine & Dentistry, The University Dodoma, Dodoma, Tanzania; 2 Department of Internal Medicine, The Benjamin Mkapa Hospital, Dodoma, Tanzania; 3 Department of Cardiology, The Benjamin Mkapa Hospital, Dodoma, Tanzania; 4 Department of Psychiatry and Mental Health, School of Medicine, The University Dodoma, Dodoma, Tanzania; UN Mehta Institute of Cardiology and Research Center, INDIA

## Abstract

**Introduction:**

Stroke survivors develop cognitive impairment, which significantly impacts their quality of life, their families, and the community as a whole but not given attention. This study aims to determine the incidence and predictors of post-stroke cognitive impairment (PSCI) among adult stroke patients admitted to a tertiary hospital in Dodoma, Tanzania.

**Methodology:**

A prospective cohort study was conducted at tertiary hospitals in the Dodoma region, central Tanzania. A sample size of 158 participants with the first stroke confirmed by CT/MRI brain aged ≥ 18 years met the criteria. At baseline, social-demographic, cardiovascular risks and stroke characteristics were acquired, and then at 30 days, participants were evaluated for cognitive functioning using Montreal Cognitive Assessment (MoCA). Key confounders for cognitive impairment, such as depression and apathy, were evaluated using the Personal Health Questionnaire (PHQ-9) and Apathy Evaluation Scale (AES), respectively. Descriptive statistics were used to summarise data; continuous data were reported as Mean (SD) or Median (IQR), and categorical data were summarised using proportions and frequencies. Univariate and multivariable logistic regression analysis was used to determine predictors of PSCI.

**Results:**

The median age of the 158 participants was 58.7 years; 57.6% of them were female, and 80.4% of them met the required criteria for post-stroke cognitive impairment. After multivariable logistic regression, left hemisphere stroke (AOR: 5.798, CI: 1.030–32.623, *p* = 0.046), a unit cm^3^ increase in infarct volume (AOR: 1.064, 95% CI: 1.018–1.113, *p* = 0.007), and apathy symptoms (AOR: 12.259, CI: 1.112–89.173, *p* = 0.041) had a significant association with PSCI.

**Conclusion:**

The study revealed a significant prevalence of PSCI; early intervention targeting stroke survivors at risk may improve their outcomes. Future research in the field will serve to dictate policies and initiatives.

## Introduction

Stroke is the leading cause of death and disability, affecting around 67 million people globally each year, with roughly 5,700,000 dying and 5,000,000 rendered incapacitated [1,2]. Stroke survivors endure cognitive impairment, which has a substantial impact on the quality of life of the sufferer, the family, and the community as a whole. PSCI is associated with reduced quality of life, increased likelihood of depressive symptoms, high level of dependence, increased health care cost, lost wages, and social isolation [3–6].

Globally, PSCI prevalence ranges from 35 to 92% [7–9]. In the few studies undertaken in Sub-Saharan Africa, 40% and 34% of Nigerian and Ghanaian stroke survivors, respectively, were diagnosed with PSCI at three and two years [10,11]. The disparity in prevalence may be rooted in variances in the diagnostic tools used to evaluate PSCI across studies, the timing of cognitive impairment screening following a stroke, ethnicity, and cultural backgrounds [12].

Ageing, female gender, fewer years of formal education, hypertension, diabetes, dyslipidaemia, atrial fibrillation, current alcohol and tobacco use, type of stroke, structures involved in stroke, stroke laterality, the size of the infarct or hematoma, and neuropsychiatric manifestations at baseline have all been linked in previous studies as independent risk factors for PSCI at a different stage of stroke [13–17]. The study aimed to assess the incidence and predictors of PSCI in early phase following a first episode of stroke among patients admitted at tertiary hospitals in Dodoma, Tanzania.

## Material and methods

### Study design and setting

This prospective cohort study was carried out at Dodoma Referral Regional Hospital and Benjamin Mkapa Hospital, both of which serve 20–30 stroke patients per month. Both are recognised teaching hospitals for the University of Dodoma for medical training at the undergraduate and residency levels. With its well-built and state-of-the-art infrastructure, the Benjamin Mkapa Hospital is equipped with neuroimaging services, such as Computed Tomography scans and Magnetic Resonance Imaging.

### Sample size and sampling procedure

The sample size was determined using a method for proportion in a prospective cohort study [18]. The estimated sample size was 130, at the very least. However, with a 30% attrition rate in our setting, 170 participants were required to meet the expected sample size. From June 2021 to March 2022, 158 participants who were willing to participate and met the inclusion criteria were recruited for the nine-month study [19].

### Inclusion criteria/exclusion criteria

Patients who were 18 years of age or older, who provided informed consent or proxy consent from a close relative if the patient is incapable, presented with their first stroke within 14 days, and whose stroke was verified by a CT scan or MRI of the brain, were included in the study. Patients with severe motor impairment on their dominant side and those with intracerebral haemorrhage from a tumour or trauma were excluded, as were those with severe sensory impairment (blindness and deafness), Transient Ischemic Attack, subarachnoid haemorrhage, and prior neurological conditions including epilepsy.

### Outcome variable

Post-stroke cognitive impairment was defined as a MoCA score of less than 23 out of 30 assessed at 30-days post admission. Compared to the widely used 26/30 cut-off, a 23/30 cut-off provides greater diagnostic accuracy [20]. A group with lesser levels of education has proven to benefit from the MoCA tool. The tool examines eight major cognitive domains: visuospatial-executive (trail making B task, 3-D cube copy and clock drawing); naming (unfamiliar animals); language (sentence repetition and phonemic fluency task); short-term memory (delayed recall of words); abstraction (verbal abstraction); attention and calculation (digits forward and backwards, target detection using tapping, serial 7s subtraction) and orientation (time place and people) [21].

### Independent variables

Through a questionnaire that was structured based on existing evidence, variables such as age, gender, level of education, history of current /less than one year of alcohol use, cigarette smoking, and diabetes were acquired [22]. Other confounding clinical variables, such as post-stroke depression and apathy, were also assessed using the Patient Health Questionnaire (PHQ) and Apathy Evaluation Scale (AES), respectively.

Blood pressure (BP) readings were recorded according to the 2018 AHA/ACC Hypertension guideline for standard measurement of BP [23]. Hypertension was defined as BP ≥140/90 mmHg or a patient on antihypertensive medications [24]. Radial pulse and heart rate were also recorded; a deficit of ten or more was considered to indicate atrial fibrillation [25].

A blood sample was analysed for Lipid profiles; according to the National Cholesterol Education Program (NCEP), dyslipidaemia will be defined as HDL-Cholesterol <40 mg/dl or Total Cholesterol ≥200 mg/dl, or LDL-Cholesterol ≥130 mg/dl or triglyceride levels ≥130mg/dl [26]. Hyperglycaemia was defined according to the American Diabetes Association as random blood sugar >11.1 mmol/L, fasting blood sugar > 7.0 mmol/L or glycated haemoglobin≥ 6.5% [27].

A 12-lead ECG was done on each participant under the supervision of a consultant cardiologist. Atrial fibrillation was diagnosed as the absence of P waves and irregular-irregular RR interval [28]. Further screening for atrial fibrillation using a 24-hour ECG Holter was done in a patient with ischemic stroke whose 12-lead ECG tracing was normal [29].

All patients had brain imaging with either a Computed Tomography scan (SIEMENS-SOMATOM Definition Flash) or Magnetic Resonance Imaging (MAGNETUM SPECTRA A TIM +Dot System 3T). Strokes were characterised according to type, hemisphere affected, cortical or subcortical, and volume of infarct/hematoma, measured using the ellipsoid method [30,31].

The Patient Health Questionnaire (PHQ)– 9, with a total score of 27, was used to screen stroke survivors for post-stroke depression; the score was classified as minimal depression (1–4), mild depression (5–9), moderate depression (10–14), moderately severe depression (15–19), and severe depression (20–27). Apathy was evaluated using the apathy evaluation scale; a score > 38 was suggestive of apathy. A cut-off> 38 has sensitivity of 80% and specificity of 100% [32,33].

### Data analysis

For statistical analysis, data were entered on a Microsoft Excel sheet and then converted to IBM SPSS PC version 26. Continuous variables were reported as mean and standard deviation (SD) or Median and interquartile ranges; frequencies and percentages were used for categorical variables. Chi square and Mann-Whitney U test were used to determine the difference in Social-Demographic, cardiovascular risk factors, stroke characteristics, and neuropsychiatric manifestations, which are depression and apathy by post-stroke cognitive outcomes. The predictors were evaluated by binary logistic regression, and only variables that met at least a 20% (p-value≤0.2) statistical significance [34] were selected for multivariable Logistic regression analysis to determine independent predictors for post-stroke cognitive impairment. The adjusted odds ratio (aOR) and the 95% confidence interval (CI) were determined. Statistical significance was determined by a two-sided p ≤ 0.05.

### Ethical issues

After receiving ethical approval from the Directorate of Research and Publications (reference number MA.84/261/02), the Vice Chancellor’s office at the University of Dodoma granted authorisation for the study to be carried out. Later, the administrative divisions of Benjamin Mkapa and Dodoma Regional Referral Hospitals gave their respective approvals for data collection under the references AB.150/293/01/196 and EB.21/267/01/123. It was made clear to participants that their participation was completely optional and that they might withdraw at any time. Participants’ identities were changed to identification numbers in order to maintain privacy and confidentiality; however, their choice to participate had no bearing on the standard of care they received. Depressive symptoms in stroke survivors led to a referral to a psychiatrist for further evaluation and therapy.

## Results

**Fig 1:**
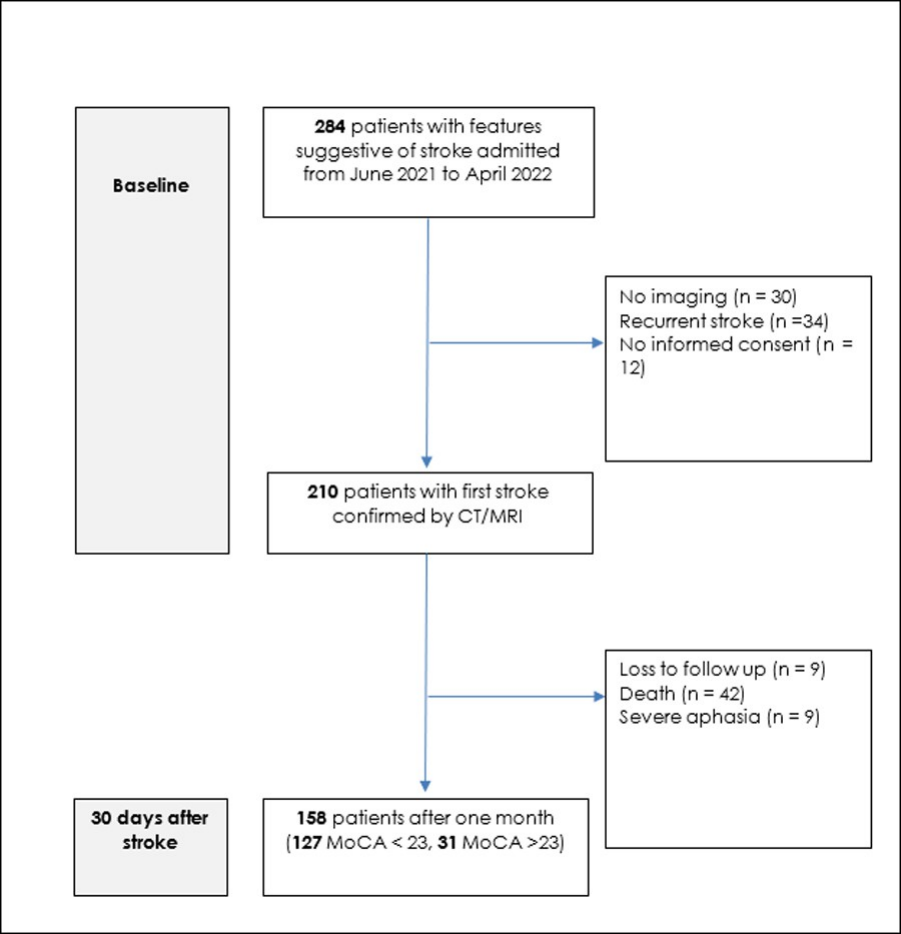
Algorithm for enrolment of study participants and 30 days post-stroke cognitive outcome.

Out of 255 stroke patients were evaluated for eligibility (Fig 1), 158 participants met the criteria and were evaluated for the Post-Stroke Cognitive Impairment at 30 days of follow-up, and 127 (80.4%) met the criteria for PSCI.

### Social demographic characteristics

The mean age of the 158 study participants was 58.7± 13.4 years, and 57.6% of them were female. The majority (66.5%) were referred from a primary healthcare facility, 50% lived in urban areas, and nearly half (49.4%) had completed seven or fewer years of formal education. Only older age (p > 0.001) and seven or fewer years of formal education (p 0.001) demonstrated significant differences with post-stroke cognitive outcomes (Tables 1 and 2).

**Table 1:** Demographic and clinical characteristics of patients with different cognitive outcomes (N = 158).

	All (N = 158)	No PSCI (N = 31)	PSCI (N = 127)	
Variables	Frequency (%)	Frequency (%)	Frequency (%)	P-value
**Social Demographic characteristics**				
Age **(Mean ± SD)**	58.7 ± 13.4	50.5 ± 12.5	60 ± 12.9	
<50	37 (23.4)	10 (32.3)	27 (21.3)	0.001
50–60	53 (33.5)	17 (54.8)	36 (28.3)	
>60	68 (43.1)	4 (12.9)	64 (50.4)	
Sex				
Male	67 (42.4)	9 (29)	58 (45.7)	0.093
Female	91 (57.6)	22 (71)	69 (54.3)	
Residence				
Urban	79 (50)	11 (35.5)	68 (53.5)	0.071
Rural	79 (50)	20 (64.5)	59 (46.5)	
Referral status				
Self	53 (33.5)	13 (41.9)	40 (31.5)	0.270
Referred	105 (66.5)	18 (58.1)	87 (68.5)	
Years of formal education				
≤ 7 years	78 (49.4)	4 (12.9)	74 (58.3)	< 0.001
≥ 8 years	80 (50.6)	27 (87.1)	53 (41.7)	
**Vascular risk factors**				
Current Cigarette smoking	33 (20.9)	5 (16.1)	28 (22)	0.467
Current Alcohol intake	27 (17.1)	1 (3.2)	26 (20.5)	0.022
Hypertension	117 (94.1)	19 (61.3)	98 (77.2)	0.071
Diabetes	36 (22.8)	7 (22.6)	29 (22.8)	0.976
Atrial fibrillation	31 (19.6)	4 (12.9)	27 (21.3)	0.294
Dyslipidaemia	106 (67.1)	20 (64.5)	86 (67.7)	0.734
**Stroke characteristics**				
Stroke type				
Ischemic	109 (69.3)	21 (67.7)	88 (69.3)	0.867
Haemorrhagic	49(30.7)	10 (32.3)	39 (30.7)	
Structures involved				
Cortical	88 (55.7)	10 (32.3)	78 (61.4)	0.003
Subcortical	70 (44.3)	21 (67.7)	49 (38.6)	
Stroke laterality				
Left	97 (61.4)	10 (32.3)	87 (68.5)	< 0.001
Right/brain stem, cerebellum	61 (38.6)	21 (67.7)	40 (31.5)	
Stroke vascular territory				
Posterior	16 (10.1)	5 (16.1)	11 (8.7)	0.217
Anterior	142 (89.9)	26 (83.9)	116 (91.3)	
**Psychiatric factors**				
Apathy	57 (36.1)	3 (9.7)	54 (42.5)	0.001
Depression				
Minimal-moderate	127 (80.4)	27 (87.1)	100 (78.7)	0.294
Severe	31 (19.6)	4 (12.9)	27 (21.3)	

**Table 2:** Clinical characteristics of patients with different cognitive outcomes (N = 158).

	All (N = 158)	No PSCI (N = 31)	PSCI (N = 127)	
Variable	Median (IQR)	Median (IQR)	Median (IQR)	P-value
**Stroke characteristics**				
NIHSS scale	12 (7)	12 (8)	12(7)	0.813
Infarct volume (cm^3^)	40 (87)	15 (25)	23 (35)	< 0.001
Hematoma volume (cm^3^)	20.7 (28)	15 (25)	23 (35)	0.248

### Clinical characteristics of participants

Thirty-one participants (19.6%) had atrial fibrillation, 36 (22.6%) were diabetic, 106 (67.1%) had dyslipidaemia, and 117 (94.1%) of the patients had hypertension. There was no significant difference in post-stroke cognitive outcomes by other vascular risk factors; however, a higher proportion (20.5%) of patients with a history of alcohol use were substantially overrepresented among stroke survivors with post-stroke cognitive impairment (p = 0.022) (Tables 1 and 2).

The majority of strokes (69.3%) were ischemic, and the median infarct and hematoma volumes were 40 and 20.7 IQR (87 and 28), respectively. Only the infarct volume, cortical strokes, and left-sided strokes exhibited significantly greater proportions among those who had post-stroke cognitive impairment (p 0.001, p = 0.003, and p 0.001, respectively) (Tables 1 and 2).

The majority of individuals (80.4%) fit the criteria for mild to moderate depression, with a median PHQ-9 score of 8, and IQR of (10), whereas apathy was found in 36.1% of participants, with a median EAS score of 34, IQR (17). Only apathy was substantially overrepresented among post-stroke cognitive impairment subjects (p 0.001) (Tables 1 and 2).

### Predictors of post-stroke cognitive impairment

Under unadjusted logistic regression, increasing age, less than eight years of formal education, hypertension, a history of current alcohol use, increasing infarct volume, left-sided stroke, cortical stroke, and apathy were all significantly associated with post-stroke cognitive impairment (Table 3). However, under adjusted logistic regression, only increasing infarct volume (AOR: 1.064, 95% CI: 1.018–1.113, *p* = 0.007), left-sided stroke (AOR: 5.798, CI: 1.030–32.623, *p* = 0.046), and apathy (AOR: 12.259, CI: 1.112–89.173, *p* = 0.041) remained significantly associated with cognitive impairment at 5% (p≤0.05) level of significance while increasing age (*p* = 0.072) had 10% level of significance (Table 2).

**Table 3:** Logistic regression analysis of predictors of cognitive Impairment at 1 month.

	Unadjusted results		Adjusted results	
Variable	OR (95% CI)	P-value	AOR (95% CI)	P-value
Age	1.064 (1.028–1.101)	< 0.001	1.075 (0.993–1.163)	0.072
Male gender	2.055 (0.878–4.810)	0.097	0.773 (0.170–3.525)	0.740
< 8 Years of formal education	9.425 (3.113–28.532)	< 0.001	2.802 (0.510–15.399)	0.236
Cigarette smoking	1.471 (0.517–4.182)	0.469		
Alcohol use	4.636(0.593–36.260)	0.144	6.858 (0.470–72.067)	0.159
Hypertension	2.134 (0.928–4.910)	0.074	0.936 (0.162–5.395)	0.941
Diabetes	1.015 (0.397–2.593)	0.976		
Dyslipidaemia	1.154 (0.506–2.631)	0.734		
Atrial fibrillation	1.822 (0.587–5.658)	0.299		
NIHSS	1.014 (0.932–1.102)	0.753		
Stroke type, Ischemic	1.074 (0.463–2.494)	0.867		
Infarct volume	1.048 (1.019–1.078)	0.001	1.064 (1.018–1.113)	0.007
Hematoma volume	1.026 (0.979–1.074)	0.288		
Stroke laterality, Left	4.002 (1.764–9.081)	0.001	5.798 (1.030–32.623)	0.046
Structures involved, Cortical	3.343 (1.453–7.693)	0.005	1.057 (0.131–8.540)	0.959
Apathy	6.904 (1.995–23.895)	0.002	12.259 (1.112–89.173)	0.041
Depression	1.558 (0.492–4.931)	0.451		

## Discussion

The main objective of this study was to determine the predictors of early cognitive impairment among patients with first-ever stroke admitted at tertiary hospitals in Dodoma. Moreover, we also determined the prevalence of post-stroke cognitive impairment. We revealed a high prevalence of PSCI at 30 days (80.4%), which was independently associated with stroke laterality, increasing infarct volume and apathy.

While the prevalence of PSCI varies around the globe, our findings allude to the high incidence and prevalence of PSCI in the early stages after a stroke episode, observed in past studies [34]. The PSCI rates generally range from 20–70% depending on the definition, phases of the stroke, severity of the stroke at admission, population heterogeneity, and pre-morbid cognitive functioning [7–9]. Similarly, high PSCI rates of 66.4–75.2% are reported when cognitive assessment is done at a comparable time frame of two to eight weeks after the stroke [7,9,35]. However, a lower prevalence of 57 and 67% was observed in the acute phase among individuals without pre-morbid cognitive impairment [36]. In general, using screening tools for evaluation of cognitive functioning shows a higher prevalence of PSCI, as observed in this study; on the contrary, when a comprehensive neuropsychological battery is used, prevalence as low as 34% and 39% were reported in Ghana and Nigeria, respectively [10,11]. Higher rates of PSCI could further be explained by the significant proportion of our study participants having less than seven years of formal education and residing in rural areas; these two factors are shown to be independent predictors of poor performance on cognitive functioning in the previous studies and also supported by our findings [10].

The association between post-stroke cognitive impairment and left hemisphere stroke observed in this study is the replication of previous findings [34,37]. Since language is primarily a left hemispheric cognitive domain for more than 90% of individuals globally [38], damage to the left hemisphere due to stroke could significantly impact the language domain and overall cognitive performance [39].

The index study showed that every (cm^3^) unit increase in infarct volume predicted PSCI; the link between a larger infarct volume and PSCI was initially described by Tomlison et al., who demonstrated that infarct volume closer to 100 cm3 considerably increased the likelihood of PSCI [40]. Kumral et al. demonstrated that infarct volume over 90 cm^3^ independently predicted PSCI [41]. The correlation between the infarct volume and PSCI has been shown in earlier studies; an infarct greater than 17 cm3 may be adequate to predict PSCI independently [34]. However, in the multitude of methodological approaches to measuring infarct volumes in the aforementioned studies, predicting PSCI based solely on infarct volume parameters needs more evidence to improve the reliability [42].

Several neuropsychiatric phenomena, including apathy, may share a common pathway to PSCI; based on the strong correlation, apathy may be considered an inherent sign of cognitive impairment rather than a distinct neuropsychiatric condition [14,43]. The same underlying brain lesion may drive apathy and cognitive impairment, specifically, the frontal lobes and subcortical structures, where the corresponding lesions may lead to the loss of cognitive function that restricts a person’s ability to organise goal-directed behaviour [44].

Given the high risk and debilitating complications with profound disabilities among stroke survivors, early stratification of those at risk for cognitive impairment is highly recommended [45–47]. Identifying patients who could benefit from early cognitive assessment is crucial for better outcomes through somatic and psychological interventions [48].

### Limitation of the study

This prospective cohort study design had a high attrition rate due to loss to follow-up and death; this needed extensive recruitment of patients to mitigate the effect. Since the pre-morbid cognitive assessment was not assessed, we could not clearly understand the status of pre-stroke cognitive functions; hence, its influence on PSCI remains speculative. Therefore, a survey such as an Informant Questionnaire for Cognitive Decline in the Elderly (IQCODE) [49] may be included in research designs to collect baseline data for pre-stroke cognitive performance.

The exclusion of patients with TIA may be confounding since TIA may raise the risk of cognitive impairment in at least one cognitive domain by approximately 30% [50], underscoring the benefits of screening cognitive changes in minor cerebrovascular [51]. Similarly, using MoCA rather than the gold standard test (comprehensive neuropsychological battery) limited the diagnostic accuracy, grading the severity of cognitive impairments, determining functional limitations, and planning for ideal treatment and rehabilitation [52].

## Conclusion

Post-stroke cognitive impairment is a common manifestation among stroke survivors in the early phase of recovery. Factors associated with PSCI are predictable; thus, identifying and targeting individuals at risk for specific interventions in the acute setting is crucial. For a comprehensive understanding of the magnitude, drivers, characteristics and overall clinical course of PSCI, well-designed long-term prospective research, including clinical trials, is necessary for progress.

## Supporting information

S1 Checklist STROBE statement—checklist of items that should be included in reports of observational studies. (DOCX)

S1 File IRB approval for data collection. (PDF)

S2 File Psci excel deidentified data. (XLSX)
